# A review of the public sector substance use disorder treatment and prevention systems in Kenya

**DOI:** 10.1186/s13011-020-00291-5

**Published:** 2020-07-20

**Authors:** Florence Jaguga, Edith Kwobah

**Affiliations:** Moi Teaching & Referral Hospital, P.O. BOX 3-30100, Eldoret, Kenya

**Keywords:** Substance use disorder, Treatment, Prevention, Systems

## Abstract

**Background:**

The burden of substance use disorders in sub-Saharan Africa has been projected to increase by an estimated 130% by 2050. Despite this, little is known about the substance use disorder treatment and prevention systems in the region.

**Objectives:**

The objective of this review is to describe the public sector substance use disorder treatment and prevention systems in Kenya guided by the World Health Organization health systems framework model, with the aim of informing decision-making.

**Methods:**

We reviewed official government documents obtained from hand-searching the websites of relevant governmental organizations including: Ministry of Health, National Authority for the Campaign Against Alcohol and Drug Abuse, Parliament of Kenya, Ministry of Treasury & National Planning, National Law Reporting Council, Kenya National Bureau of Statistics, the National Non-Governmental Organization (NGO) Coordination Board and the 47 County Governments. We augmented those searches with official documents that the authors were aware of by virtue of being practitioners in the field. Draft and retired documents were excluded. The findings of the search are presented as a narrative review.

**Discussion:**

The *Mental Health Act 1989,* the main legislative framework governing substance use disorder treatment and prevention, focuses on institutional care only. While there are only three public health facilities offering substance use disorder treatment in Kenya, several non-public sector actors are involved in SUD treatment and prevention activities. Unfortunately, there is limited cross-sector collaboration. The Ministry of Health has no specific budget for substance use disorder treatment and prevention, while the National Authority for the Campaign Against Alcohol and Drug Abuse has an annual resource gap of about US$ 5,000,000. The substance use disorder workforce in Kenya has not been characterized.

**Conclusion:**

We propose five key strategies for strengthening substance use disorder treatment and prevention systems in Kenya including: (1) Enactment of the *Mental Health (Amendment) bill 2018.* (2) Integration of substance use disorder treatment and prevention into primary health care to increase access to care. (3) Utilization of money from taxation of alcohol, tobacco and betting to increase funding for substance use disorder treatment and prevention. (4) Characterization of the substance use disorder workforce to inform planning. (5) Enhanced collaboration between the government and non-state actors in order to increase access to SUD treatment and prevention.

## Background

The burden of substance use disorders (SUDs) in sub-Saharan Africa has been projected to increase by an estimated 130% by 2050 [1]. Despite this, SUD treatment and prevention systems in the region remain under-resourced [2] and the treatment gap is as high as 87% [3]. Untreated SUDs are a major public health problem. Globally, SUDs are the second leading cause of disability among the mental disorders with 31,052,000 (25%) Years Lived with Disability (YLD) attributed to them [4]. In Africa, the impact of problematic patterns of substance use is particularly high. For example in 2016, despite lower levels of alcohol consumption compared to elsewhere, the region had the highest age-standardized alcohol-attributable deaths and age-standardized alcohol-attributable Disability Adjusted Life Years (DALYs) (70.6 deaths per 100,000 people and 3043.7 per 100,000 people respectively) [5]. Further compounding this situation, is the fact that SUDs are associated with social costs as high as I$ 800 per head [6], emanating from their impact on productivity, crime and health systems [7]. In sub-Saharan Africa where the burden of SUDs is high and countries are struggling to end poverty, it is important that governments place priority on strengthening their SUD treatment and prevention systems.

The World Health Organization (WHO) defines health systems strengthening as the process of improving the six health system building blocks namely service delivery, workforce, health information, medical products, financing and governance in order to achieve optimal health outcomes [8]. A well-functioning health system is therefore one that provides services that are safe, accessible and of quality; has a competent workforce that is sufficient in number; produces and disseminates health information in a timely manner; provides essential medication that is affordable; allocates adequate funds to health and finally is guided by strategic policies [8]. In Kenya, the need for an effective SUD treatment and prevention system is pressing. Over 10% of Kenyans aged between 15 and 65 years have an alcohol use disorder, with most of them (60%) having the severe form [9]. In fact, the country has one of the highest total DALYs (54,000) from alcohol use disorders in Africa [10]. Moreover, early onset substance use is a significant problem. In 2019, one in five adolescents were reported to have ever used at least one substance in their lifetime [11]. In that survey, the median age of onset was 11 years [11] and considerably lower than that (16–19 years) reported elsewhere in the world [12]. Among both adults and adolescents in Kenya, substance use has been linked to risky sexual behavior [11, 12] which is a major driver of Human Immunodeficiency Virus (HIV) transmission in Kenya [13].

Unfortunately, the existing SUD treatment and prevention systems in Kenya are inadequate. The main legislative framework governing SUD treatment and prevention, the *Mental Health Act 1989* [14] is outdated and focuses on institutional care. Three out of over 5000 government run health facilities, deliver treatment for SUDs [15]. Moreover, the Ministry of Health (MOH) has no separate budget for SUD treatment and prevention. The objectives of this review are: (i) to provide an overview of the current state of public sector SUD treatment and prevention systems in Kenya guided by the WHO health systems framework. Prior literature examining the subject in sub-Saharan Africa is scarce and has mainly focused on select health system components [2, 16] (ii) to suggest recommendations for improvement. Such information could be useful in guiding the strengthening of SUD treatment and prevention systems in Kenya in line with Vision 2030, the country’s long-term development blueprint [17], and target 3.5 of the Sustainable Development Goals (SDGs) [18].

## Methods

Since our aim was to describe the public sector SUD treatment and prevention systems, we reviewed official government documents. We hand-searched the websites of relevant governmental organizations for documents and web pages that had content on SUD treatment and prevention. The organizations whose websites were searched include: MOH, National Authority for the Campaign against Alcohol and Drug Abuse (NACADA), Parliament of Kenya, National Council for Law Reporting, Kenya National Bureau of Statistics (KNBS), National Non-Governmental Organization (NGO) Coordination Board, each of the 47 County Governments.^1^ We excluded draft documents and those that had been replaced by newer versions. We augmented documents obtained from the searches with those that the authors were aware of by virtue of being practitioners in the mental health field. We reviewed a total of 40 documents (Table 1). The findings of the search are presented as a narrative review.

**Table 1:** Documents included in this review

Author	Document & Year
National Law Reporting Council	Mental Health Act 1989 [14]
	Alcoholic Drinks Control Act [20]
	Narcotic Drugs and Psychotropic Substances Act [21]
	Health Act 2017 [22]
	Tobacco Control Act 2007 [23]
NACADA	NACADA Strategic Plan 2019–2022 [24]
	Knowledge Attitude Practices of Christian Faith Based Organizations on Alcohol and Drug Abuse [25]
	Effectiveness of Community Based Interventions to Mitigate Harmful Alcohol Use in Murang’a East District [26]
Parliament of Kenya	National Programs Based Budget 2019/2020 [27]
	Mental Health (Amendment) Bill 2018 [28]
National Non-Governmental Organization Co-ordination Board	Annual NGO Sector Report 2018/19 [29]
Ministry National Treasury & Planning	Vision 2030 Third Medium term Plan 2018–2022 [17]
Ministry of Health	Kenya Essential Medicines List 2019 [30]
	The Kenya Mental Health Policy 2015–2030 [31]
	The National Protocol for Treatment of Substance Use Disorders in Kenya 2017 [32]
	Health Indicator and Standard Operating Procedures Manual 3rd Edition, 2017 [33]
Kenya National Bureau of Statistics	Kenya Demographic Health Survey 2014 [34]
	Kenya STEPwise Survey for Non-communicable Diseases Risk Factors 2015 Report [35]
	Global Adult Tobacco Survey 2014 [36]
Embu County Government	Embu County MTEF^a^ Programs Based Budget 2019/2020 [37]
Kisii County Government	Kisii County Programs Based Budget 2019/2020 [38]
Bomet County Government	Bomet County MTEF^a^ Programs Based Budget 2019/2020 [39]
Kajiado County Government	Kajiado County Programs Based Budget 2019/20 [40]
Kericho County Government	Kericho County Programs Based Budget 2019/2020 [41]
Laikipia County Government	Laikipia County Programs Based Budget 2019/2020 [42]
West Pokot County Government	West Pokot County Programs Based Budget 2019/2020 [43]
Elgeyo Marakwet Government	Elgeyo Marakwet County Programs Based Budget 2019/2020 [44]
Turkana County Government	Turkana County Programs Based Budget 2019/2020 [45]
Makueni Government	Makueni County Programs Based Budget 2019/2020 [46]
Machakos County Government	Machakos County Programs Based Budget 2019/2020 [47]
Kakamega County Government	Kakamega County Programs Based Budget 2019/2020 [48]
Mombasa County Government	Mombasa County Programs Based Budget 2019/2020 [49]
Vihiga County Government	Vihiga County MTEF^a^ Programs Based Budget 2018/19–2020/21 [50]
Kwale County Government	Kwale County Programs Based Budget 2018/19–2020/21 [51]
Nyeri County Government	Nyeri County Programs Based Budgets 2019/20 [52]
Lamu County Government	Lamu County MTEF^a^ Programs Based Budget 2017/18–2019/20 [53]
Samburu County Government	Samburu County Programs Based Budgets2019/2020 [54]
Wajir County Government	Wajir County Programs Based Budget 2019/2020 [55]
Kilifi County Government	Kilifi County Programs Based Budget 2019/2020 [56]
Tharaka-Nithi County Government	Tharaka-Nithi County Programs Based Budget 2019/2020 [57]

^a^*MTEF* Mid-Term Expenditure Framework

### Legislation & policy governing SUD treatment and prevention in Kenya

The Vision 2030 [17], Kenya’s overall national development policy framework, recognizes SUDs as barriers to the attainment of national transformation, education, gender equality and the well-being of youth and other vulnerable populations. In doing so, the policy requires that the government implements projects and programs to mitigate the harmful impact of SUDs.

Unfortunately, the *Mental Health Act, 1989* [14] which provides the main legal framework for addressing SUD treatment and prevention in Kenya is outdated and not in line with Vision 2030. The act focuses on institutional care and has no provisions mandating the delivery of preventive, rehabilitative or community based care. Further, the act does not address critical issues such as access to care, stigma, discrimination and the rights of persons who use mental health and SUD services [58]. Moreover, the act is at odds with the current *Constitution of Kenya, 2010* which devolves health functions to the county governments [19]. The *Mental Health Act, 1989* [14] however recognizes a SUD as a mental disorder in line with the current understanding of these disorders as health problems [59].

The proposed *Mental Health (Amendment) Bill, 2018* [28], currently awaiting its first reading in parliament seeks to overhaul the *Mental Health Act, 1989* [14]. In addressing the limitations of this Act, the bill requires that the government provides community based care. It directs that treatment and prevention of mental health and SUDs includes prevention, early intervention, rehabilitation and follow-up. The bill additionally contains provisions that mandate the government to ensure access to care, to promote non-discriminatory practices by insurance providers, and to ensure that the rights of persons with mental health conditions and SUDs are upheld. In line with the *Constitution of Kenya, 2010* [19], the bill delineates the roles and responsibilities of the National and County Governments in mental health and SUD service delivery.

Importantly, this bill is aligned with Vision 2030 [17], and guidelines for the delivery of SUD services presented in the Kenya Mental Health Policy 2015–2030 [31]. The Kenya Mental Health Policy 2015–2030 recommends that in treating SUDs, services should be evidence-based, comprehensive and universally accessible. The policy recommends the establishment of preventive programs, community based services, and the incorporation of psychosocial rehabilitation into SUD treatment. In order to ensure accessibility, the policy recommends that treatment for SUDs be provided at all levels of the health care system [31]. Upon enactment and implementation, the *Mental Health (Amendment*) *Bill, 2018* [28] will therefore be useful in streamlining and transforming SUD treatment and prevention in Kenya.

Other than the *Mental Health Act 1989*, several other laws contain provisions that seek to address SUD treatment and prevention in Kenya. *The Health Act, 2017* [22]***,****The Tobacco Control Act, 2007* [23]*, The Alcoholic Drinks control Act, 2010* [20] *and The Narcotic Drugs & Psychotropic Substances (Control) Act, 1994* [21] all mandate the government to educate the public on the adverse health consequences of substances. They further require that the Ministry of Education and the MOH integrate instruction on the health consequences posed by substances into syllabuses and healthcare respectively.

*The Alcoholic Drinks Control Act, 2010* [20] *and* the *Narcotic Drugs & Psychotropic Substances, Act 1994* [21] specifically mandate that the government provides rehabilitation programs for persons dependent on alcohol and illegal substances respectively. The *Narcotic Drugs & Psychotropic Substances Act,* 1994 [21] provides for committal to rehabilitation for those found in possession if the court is satisfied that the person arrested is addicted and was in possession for his own personal consumption. Both Acts require that the government sets up a fund consisting of monies obtained from licensing fees, fines and grants for purposes of SUD treatment and prevention. Currently part of the funds obtained under the *Alcoholic Drinks Control Act 2010* [20] are allocated to the NACADA and to civil society organizations conducting SUD programs to facilitate their operations [24, 60].

Unfortunately, The *Alcoholic Drinks Control Act 2010* [20] *and* the *Narcotic Drugs & Psychotropic Substances Act 1994* [21] mandate punitive measures for substance use and possessions, and lack a human rights approach. Persons who use substances therefore frequently get incarcerated and exposed to harassment, discrimination and human rights abuses by law enforcement [61]. In a joint statement in 2017, the United Nations (UN) and the WHO recommended the reviewing and repealing of laws that criminalize substance use or possession of substances for personal use [62]. This has not yet been done in Kenya.

Workplace policies on SUD treatment and prevention are an integral part of SUD management at the institutional level and are an International Labor Organization (ILO) requirement [63]. Such policies which offer guidelines on the strategies and measures to be undertaken in order to mitigate the harmful impact of SUDs in the workplace, are currently not mandated by existing local legislation. The NACADA has however published guidelines for the development of workplace policies on SUD treatment and prevention that organizations can refer to in preparing their own policies [64].

### SUD treatment and prevention: service delivery

SUD treatment and prevention services are delivered within the health sector by the MOH, and the County Government Departments of Health; and outside the health sector by the NACADA and various County Government Departments.

#### Health sector SUD service delivery

The key responsibilities of the MOH include developing health policies, managing five national referral health facilities as well as oversight of service delivery at the County level. The County Departments of Health are in turn tasked with the delivery of facility and community based health care at the county level, and report to the MOH [65]. In realization of its mandates, the MOH through its Division of Mental Health and Substance Abuse Management, has successfully spearheaded the development of a treatment protocol for SUDs [32] and the Kenya Mental Health Policy 2015–2030 [31]. The MOH additionally supports and co-ordinates opiate substitution therapy and needle exchange programs as HIV prevention interventions through its National AIDS and STI’s Control Programme. This is largely done in collaboration with NGOs [66, 67].

The health sector SUD services however remain scarce and inaccessible. Out of 5800 health facilities run by the MOH and County Departments of Health in Kenya, only three offer SUD rehabilitation services and all are located in urban centers [15]. Further, only three County Health Departments had planned programs relating to SUD treatment and prevention for the 2019/2020 financial year (Table 2). The opiate substitution therapy clinics also located in urban areas, are heavily supported by donor funding [67] and their sustainability therefore remains a challenge.

**Table 2:** County Government SUD treatment and prevention programs

	County	Program description (Based on Programs Based Budget 2019/2020)	County Departments supervising the program	Amount allocated (US$)
1.	Kakamega [48]	Alcohol and drug abuse Rehabilitation program (construction and purchase of equipment)	Department of Public Service and administration	54,905^a^
2.	Mombasa [49]	Control of drug and substance abuse, Medically Assisted Therapy	Department of Health services	225,884^b^
3.	Samburu [54]	Increase awareness on alcohol and drug abuse	Department of Health services	3,442,039^c^
4.	Kwale [51]	Management of drug and substance abuse rehabilitation centre	Department of Social services and talent management	32,366
5.	Kilifi [56]	Campaign and sensitization against drug/substance abuse	Department of Gender, Culture, Social Services and Sports	40,000^d^
6.	Embu [37]	Rehabilitation of persons with SUDs	Department of Youth empowerment, gender, children, culture and social services	81,061
7.	Machakos [47]	Create awareness on SUDs	Department of Tourism, culture, Youth, Sports	13,308
8.	Makueni [46]	Anti-drug & substance abuse program	Department of Education, Youth, Sports, and Information Communication and Technology	300,000^e^
9.	Turkana [45]	Rehabilitation and treatment for alcohol and drug abuse; Public education advocacy and awareness	Department of Health services and sanitation	19,000
10.	Elgeyo Marakwet [44]	Baseline survey on prevalence of alcohol use	Department of Public Administration and governance	556,040^f^

^a^amount includes allocation for alcoholic drinks control

^b^amount includes allocation for family, maternal, adolescent and child health; Malaria; Tuberculosis treatment and prevention; HIV/AIDS treatment and prevention

^c^amount was allocated to ‘preventive and promotive health services’ with ‘increasing awareness on alcohol and drug abuse’ as one of the programs

^d^amount includes allocation for anti-radicalization, peace and security campaigns; youth talent identification; youth economic empowerment, campaign against teenage pregnancy and youth skills training

^e^amount includes allocation for sports development

^f^amount includes allocation for inspection of alcoholic drinks outlets

#### Role of the NACADA

The NACADA is a State Corporation in the Ministry of Interior and Coordination of National Government. Its role centers on SUD prevention [68]. The NACADA therefore runs a series of public education programs often in collaboration with other public and private organizations [68]. The authority which has one national and eight regional offices across the country, recently piloted a life skills training program in 81 primary schools across the country in order to curb early substance use [24]. Other than public education, the NACADA is responsible for licensing and regulating SUD rehabilitation facilities [69].

#### Other County departments

In the 2019/2020 financial year, 70% of the Counties had their SUD programs under various departments including Public administration, Education, Social services and Youth empowerment (Table 2). It has been recommended that SUD treatment and prevention be integrated into mainstream health care as this ensures concurrent management of comorbidities, increased access to services as well as adequate quality of care [70].

#### Role of non-state actors

In order to ensure improved access to SUD treatment and prevention, as well as achieve efficiencies in service delivery, there is need for collaborative and complementary work between the government and non-state actors. Based on data from the NACADA, there are more than 80 accredited private-for-profit SUD rehabilitation facilities across the country [15]. Private sector healthcare is however costly and therefore inaccessible for a majority of Kenyans [71]. A number of NGOs are involved in SUD treatment and prevention activities. In the 2018/2019 financial year, 4% of newly registered NGOs implemented projects on SUD treatment and prevention. During the same period, a 51% increase in utilization of funds on SUD programs was noted compared to the prior financial year, indicating growing NGO involvement in the field [29]. A lack of meaningful engagement has been reported in Kenya between the government on one hand and the private sector and NGOs on the other [29, 72].

The involvement of faith based organizations and community based organizations in delivering SUD prevention has been documented in Kenya [25, 26]. The activities of both types of organizations largely revolve around public education but also include economic empowerment for brewers of illicit alcohol and persons with SUDs. However, such organizations often lack adequate knowledge and finances necessary for implementing impactful interventions within their spheres of influence [25, 26].

Like elsewhere in the world [73], mutual self-help groups are a part of SUD treatment in Kenya. There are over 30 Alcoholics Anonymous groups spread across several major cities in the country [74]. Participation in mutual self-help groups has been shown to lead to reduced substance consumption as well as lessen the need for more costly specialized treatment services [73].

A number of opportunities exist for meaningful collaboration between the government and non-state actors in SUD treatment and prevention in Kenya: (i) Capacity building of faith based organizations, community based organizations and mutual self-help groups by the government in order to make them potential means for SUD treatment and prevention at the grassroots level. (ii) Accreditation of private SUD treatment facilities by the National Hospital Insurance Fund (NHIF) to increase access to specialized SUD treatment. Currently, only three private rehabilitation facilities are accredited [15].

### Government budgetary allocation to SUD treatment and prevention

The MOH had no specific budgetary allocation for SUD treatment and prevention [27]. At the County level, only 10 out of 20 counties whose 2019/2020 budgets were available online had allocation to SUD treatment and prevention programs (Table 2). The exact amounts allocated to SUD programs were in most instances difficult to quantify since they had been lumped together with other programs. Where SUD programs had been budgeted for separately, allocations ranged from US$ 13,000 to 80,000 per annum (Table 2). The NACADA receives funding from the government and from the Alcoholic Drinks Control Act fund totaling to about US$ 5,000,000 annually [24]. According to its 2019–2023 strategic plan the authority will require an average of US$ 10,000,000 annually to carry out its activities. This leaves the authority with an annual resource gap of about US$ 5,000,000 [24].

The cost of interventions for alcohol use disorders has been estimated and could serve as a guide during planning. For example, public awareness campaigns to prevent harmful alcohol use have been approximated to cost US$ 0.2–0.8 per person in the population per annum, and that of brief interventions offered within the health sector US$ 0.4–1.8 per person in the population per annum [75]. Strategies to increase funding for SUD treatment and prevention have also been proposed. These include the use of money from taxation of legal substances and betting for SUD care. It has further been recommended that all financial resources for SUD care be pooled into a common fund to ensure efficient use [76]. Such strategies could be adopted in Kenya.

### SUD treatment and prevention: human resource

Globally, the SUD treatment and prevention workforce is diverse comprising of peers, teachers, counselors, social workers and nurses [15, 70]. Certification in SUD treatment and prevention has therefore been introduced as a way of standardizing care [77]. In Kenya, NACADA offers a certification course, the Basic Universal Treatment Curriculum Training Program for Addiction Professionals [69] Information on the number of certified counselors trained so far, their current placement, professional background and responsibilities was however not available. In order to make useful recommendations, the SUD workforce in Kenya needs to be characterized. Nonetheless, integration of SUD treatment and prevention into primary care has been shown to be cost-effective resulting in an estimated I$ 3,000 per DALY saved [75]. It is therefore important that primary health care workers in Kenya receive training on SUD treatment and prevention and form the frontline SUD workforce.

### SUD treatment and prevention: information systems

Kenya has a number of sources of information on substance use that could inform interventions. The KNBS in collaboration with various stakeholders conducts several national household surveys. The Kenya Demographic Health Survey collects data on the patterns of alcohol consumption among other health indicators and has been conducted twice so far (in 2003 and 2014) [34]. The Global Adult Tobacco Survey provides information on the patterns of tobacco use among persons 15 years and older [36] while the STEPwise survey for non-communicable disease risk factors, collects information on alcohol and tobacco use among other risk factors [35]. The NACADA conducts nationwide general population surveys every 5 years on the status of substance use and SUDS. Data collected includes prevalence rates as well as information on knowledge, awareness and health consequences. The authority also conducts other surveys examining the burden and impact of substance use among specific populations on a need basis [78].

The MOH through its health sector indicator manual [33] provides guidelines for the minimum set of health indicators to be collected to facilitate planning. Four of these relate to SUDs: (i) percentage of adults who are heavy episodic drinkers (ii) number of planning entities (e.g. Counties) that have a SUD management plan (iii) proportion of population who smoke cigarettes or a pipe or use other tobacco products (iv) proportion of campaigns conducted to create awareness on tobacco cessation. The MOH relies on surveys conducted by the KNBS and data from County Governments and health facilities to monitor these indicators [33]. Data collected by health facilities have particularly been found to be of poor quality hence not beneficial for decision-making [79].

### SUD treatment and prevention: essential medicines

The WHO recommends that at the minimum, health systems must have nicotine replacement therapy and methadone for the management of SUDs [80]. The Kenya Essential Medicine List (KEML) 2019 contains a wider range of drugs for the treatment of SUDs including Vitamin B, bupropion, buprenorphine, buprenorphine/naloxone, naltrexone, methadone and nicotine replacement therapy [30]. The availability of these drugs at the facility level is however problematic. An unpublished study conducted at the largest public mental health hospital in Kenya found that the facility had only 50% of the drugs listed in the essential medical list that was operational at the time. The authors also reported that stock outs were frequent with over half of the drugs being out of stock for 12 consecutive months [81].

### Limitations

A limitation of this review is that it describes the SUD treatment and prevention systems in Kenya based on documents and information that was available online. Information unavailable on the internet such as some County government budgets and implementation levels of planned programs was therefore missed out. A future study obtaining data from key informant interviews and review of hard copies of government documents, and guided by a structured tool such as the World Health Organization-Assessment Instrument for Mental health Systems (WHO-AIMS) [82] could provide a more comprehensive description of the SUD treatment and prevention systems in Kenya.

## Conclusion

The SUD treatment and prevention systems in Kenya are under-resourced. We propose five key strategies for strengthening SUD treatment and prevention systems in Kenya: (1) Enactment of the *Mental Health (Amendment) bill 2018*. (2) Integration of substance use disorder treatment and prevention into primary health care to increase access to care. (3) Utilization of money from taxation of alcohol, tobacco and betting to increase funding for substance use disorder treatment and prevention. (4) Characterization of the substance use disorder workforce to inform planning. (5) Enhanced collaboration between the government and non-state actors in order to increase access to SUD treatment and prevention.

## Data Availability

Not applicable.

## Abbreviations

AIDS: Acquired Immune Deficiency Syndrome
DALY: Disability Adjusted Life Year
HIV: Human Immunodeficiency Virus
I$: International Dollar
ILO: International Labor Organisation
KEML: Kenya Essential Medical List
KNBS: Kenya National Bureau of Statistics
MOH: Ministry of Health
MTEF: Mid-Term Expenditure Framework
NACADA: National Authority for the Campaign Against Alcohol and drug Abuse
NGO: Non-Governmental Organization
NHIF: National Hospital Insurance Fund
STIs: Sexually Transmitted Infections
SDG: Sustainable Development Goals
SUD: Substance use disorder
UN: United Nations
US$: United States Dollar
YLD: Years lived with Disability
WHO-AIMS: World Health Organization-Assessment Instrument for Mental health Systems
WHO: World Health Organization

## Footnotes

1 Kenya promulgated a new constitution in 2010, creating a decentralized system of government exercised at 2 levels i.e. the National government and 47 County governments. Each level has its defined roles and responsibilities [19].
